# Microcomputed tomographic analysis of the efficiency of two retreatment techniques in removing root canal filling materials from mandibular incisors

**DOI:** 10.1038/s41598-023-29156-0

**Published:** 2023-02-08

**Authors:** Xueqin Yang, Ye Wang, Mengzhen Ji, Yanyao Li, Hao Wang, Tao Luo, Yuan Gao, Ling Zou

**Affiliations:** 1 State Key Laboratory of Oral Diseases & National Clinical Research Center for Oral Diseases, Department of Endodontics, West China Hospital of Stomatology, Sichuan University, Chengdu, 610041 China; 2 Department of Stomatology, Sichuan Cancer Hospital & Institute, Sichuan Cancer Center, School of Medicine, University of Electronic Science and Technology of China, Chengdu, 610041 China

**Keywords:** Root canal treatment, Endodontic instruments

## Abstract

This study aimed to evaluate the removal effect of the ProTaper Next system (PTN) combined with manual nickel-titanium Hedström (MNiTiH) files or chloroform on filling materials of mandibular incisors by microcomputed tomography (micro-CT). Sixty-four extracted human mandibular incisors were finally selected and assigned to two groups (*n* = 32) based on root canal morphology after instrumentation. Two subgroups (*n* = 16) were formed based on two retreatment methods. The volume of residual filling materials (RFMs) and the surface area covered by RFMs were analyzed by micro-CT, the apical extrusion and the time taken for removing the filling materials were recorded. A smaller percentage of the volume of RFMs and less surface area covered by RFMs occurred in PTN combined with MNiTiH groups and round-shaped canal incisors (*P* < 0.05). The time taken for removing the filling materials was not significantly different in all groups (*P* > 0.05). The apical extrusion was significantly less in PTN combined with MNiTiH groups than in PTN combined with chloroform groups (*P* < 0.05). Neither of the two methods rendered root canals completely free from filling materials. PTN combined with MNiTiH could reduce the apical extrusion and remove filling materials more efficiently than PTN combined with chloroform.

## Introduction

Root canal treatment aims to reduce the intracanal bacterial content that will then allow periradicular lesions to heal. Due to the complexity of the anatomy of the root canal system and the severity of endodontic infection, the failure rate of root canal treatment reported in the literature is as high as 17%^1^. Root canal retreatment is usually the preferred option for teeth in which endodontic treatment has failed, as there is a greater opportunity to eradicate any intraradicular source of infection compared with a surgical approach^2^. Ideally, initial filling should be entirely removed in the process of root canal retreatment because the remnants containing microorganisms could compromise the effectiveness of canal disinfection using canal irrigation and intracanal medication. Therefore, the complete removal of the filling material remnants may be considered an important step for successful endodontic retreatment^3,4^.

Mandibular incisors are mostly single canal teeth with a canal configuration ranging from a round shape to an oval shape^5^. Several studies evaluated the effect of removing root canal filling materials of mandibular incisors with different removal techniques^6–8^. However, few studies have evaluated the influence of different root canal morphologies on the removal effect of filling materials. In addition, most of the retreatment methods reported in the literature use nickel-titanium rotary files^9^; in this approach, the filling material in the root canal is removed through high-speed rotation and the thermal effect caused by friction. Currently, thermal vertical compaction technology is widely used in the clinic to make well-compacted fillings, and there is no glide path for the insertion of the instrument during the retreatment process. The filling materials can only be removed after being softened by high-speed rotation and the thermal effect generated by friction.

Therefore, nickel-titanium rotary instruments in combination with organic solvents were used in some studies^8,10^, but the solvents have cytotoxic potential^11^ and can increase the difficulty of root canal cleaning^12^. Considering these factors, it is imperative to establish a glide path for root canal retreatment with a safer and more effective method.

In recent years, manual nickel-titanium Hedström (MNiTiH) files have been newly developed as large taper root canal retreatment instruments; these tools can establish a channel for the entry of nickel-titanium rotary files by screwing in filling materials and removing them. At the same time, MNiTiH files overcome the shortcomings of traditional stainless steel hand Hedström files that are too rigid to deal with curved root canals and have a high possibility of instrument separation in small-size instruments. Accordingly, chloroform is avoided, and a channel can be quickly established in dense filling materials. At present, there is no research on removing filling materials in root canals by MNiTiH files combined with nickel-titanium rotary files.

Microcomputed tomography (micro-CT) scanning is a noninvasive imaging technology with high resolution that can reconstruct the internal structure of teeth. In recent years, this technology has been widely used to assess the volume of residual filling materials (RFMs) in root canal retreatment in three dimensions^13,14^. However, the area of the root canal covered by RFMs has a significant impact on cleaning root canal walls and killing the residual infectious microorganisms in dentin tubules. The area of the root canal surface covered by RFMs is a critical parameter in evaluating the result of the removal of filling materials. To date, the use of the micro-CT technique to evaluate the area covered by RFMs has not been reported in the current literature.

The purpose of this study was to compare the efficiency of Ni–Ti rotary instruments combined with MNiTiH files or combined with chloroform on the removal of root canal filling materials in mandibular incisors by micro-CT. The null hypothesis was that no significant differences would be found in the efficiency of Ni–Ti rotary instruments combined with MNiTiH files or combined with chloroform on the removal of initial filling.

## Methods

### Specimen selection and canal preparation

In this study, 108 extracted human mandibular incisors were collected with the approval of the Medical Ethics Committee of West China Stomatological Hospital, Sichuan University (WCHSIRB-D-2020–388). Informed consent was obtained from patients and the study was performed in accordance with the Declaration of Helsinki. After the initial micro-CT scanning (μCT-50; Scanco Medical; Bassersdorf; Switzerland), which was performed with an isotropic resolution of 24 μm at 90 kV, 88 μA, and 8 W, mandibular incisors with fractures, two canals, or calcified canals or canals with curvatures higher than five degrees as described by Siqueira^15^ were excluded. Finally, 76 mandibular incisors with a length of 20–22 mm were selected, and the parameters of each subsequent scan were the same. All data were exported in DICOM format. These teeth were stored in 0.02% buffered thymol solution throughout the study. For all samples, a standard coronal access preparation was performed, and the working length under the microscope was established with a size 10 K-file that was 0.5 mm short of the anatomical apical foramen (Pico; Carl Zeiss; Jena; Germany). Then, teeth were embedded into rubbed silicon to simulate clinical working conditions. Root canals were prepared to X3 according to the ProTaper Next (PTN) (Dentsply Maillefer; Tulsa; Switzerland) instrument system instructions, and they were irrigated with 2 mL of 2.5% NaOCl and 17% ethylene-diamine-tetra-acetic acid (EDTA) during canal instrumentation. A final flush with 5 mL distilled water was inserted into the root canal and ultrasonically activated for 1 min. Then, the samples were rescanned using the aforementioned parameters.

### Root canal morphology grouping and obturation

According to the cross-sectional anatomy of the instrumented root canal, the samples were divided into two groups according to the aspect ratio. The canals were classified as oval-shaped canals when the major buccolingual diameter of the canal was at least twice as wide as the mesiodistal diameter throughout the coronal two-thirds (5 mm from the apex)^8,16^. In contrast, the canal was considered to have a round shape if the aspect ratio was less than 1.5^17^. Following measurement and grouping, 32 teeth with regular, round-shaped canal cross-sections were categorized into the round-canal mandibular incisor group, and 32 teeth with oval canal cross-sections were categorized into the oval-canal mandibular incisor group. Subsequently, the root canals were filled with gutta-percha points (30#/0.06) and AH-Plus sealer (Dentsply DeTrey; Konstanz; Germany) using the continuous wave condensation technique (B&L Biotech; Ansan-si; Korea). The access preparations were sealed with Caviton (GC Corporation; Tokyo; Japan). All specimens were stored in a humidor at 37 °C and 100% humidity for 4 weeks to allow filling materials to set completely.

### Grouping and root canal retreatment

After 4 weeks, a third micro-CT scan was performed. To create a similar sample distribution in the groups, sixteen anatomically pair-matched teeth were selected in each group based on similar morphological features of the canal, such as length, volume, aspect ratio, and 3-dimensional configuration.

**Figure 1:**
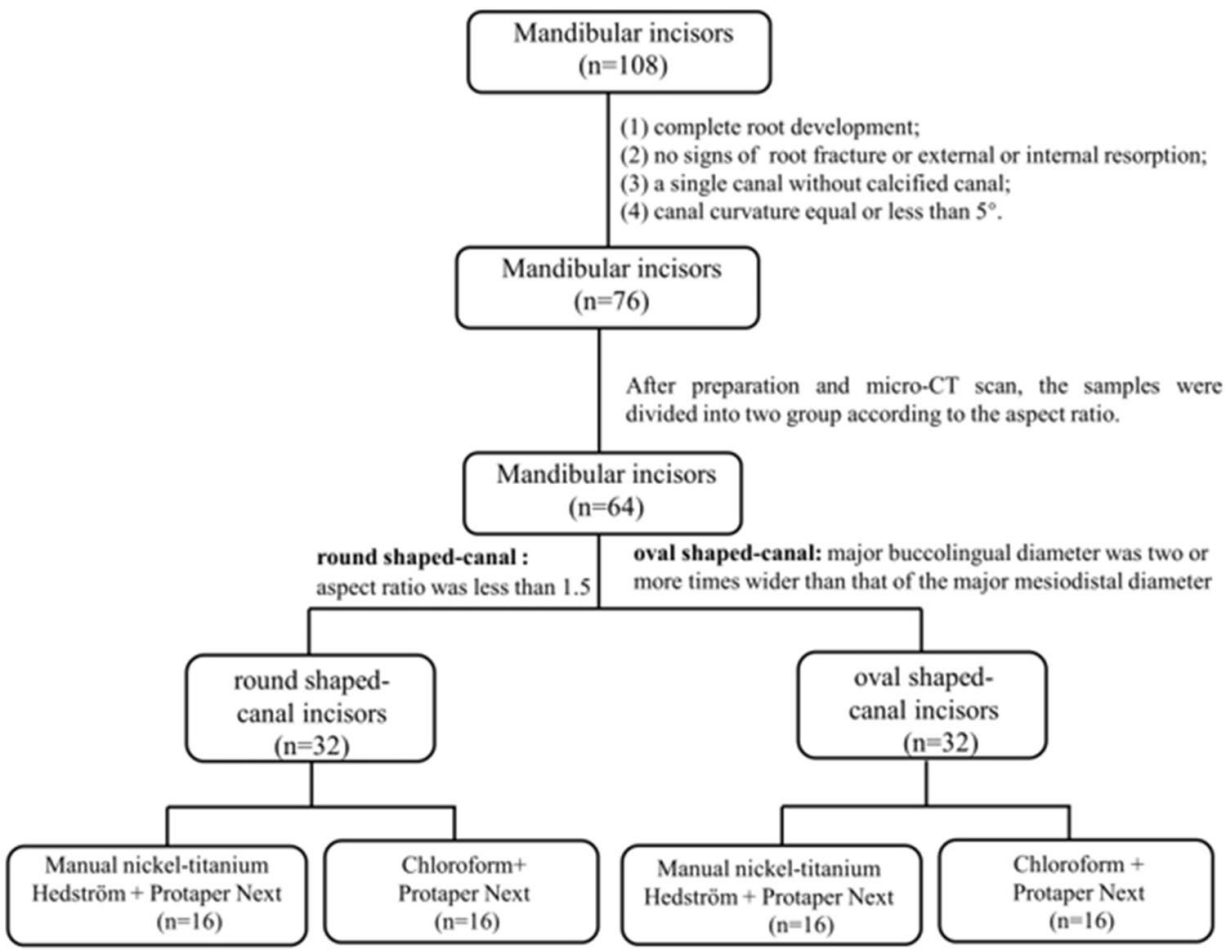
Sample screening and grouping flow diagram.

The samples were organized into four subgroups (*n* = 16) according to the filling removal technique as described below (Fig. 1).

#### Group one

R-PTN- MNiTiH (round-canal mandibular incisor: PTN combined with MNiTiH file).

#### Group two

O-PTN- MNiTiH (oval-canal mandibular incisor: PTN combined with MNiTiH file).

#### Group three

R-PTN-CL (round-canal mandibular incisor: PTN combined with chloroform).

#### Group four

O-PTN-CL (oval-canal mandibular incisor: PTN combined with chloroform).

The root was inserted into an experimental model to collect extruded apical debris during the retreatment procedure, which was described by Kocak et al*.*^18^. After removing the temporary sealing materials, the samples were treated as follows:

### PTN combined with MNiTiH file (PTN-MNiTiH)

A set of MNiTiH files (Superline NIC Dental; Shenzhen; China) contains sizes of 50#0.05, 40#0.03, 35#0.04, 30#0.03, 25#0.04, and 20#0.03. In our retreatment procedure, first, an MNiTiH file with a large taper of 40#0.03 was screwed into the filling material for 2–3 mm and met resistance. Then, the filling material was pulled out of the root canal by clamping the handle of the hand file with hemostatic forceps. If the filling material or the file could not be pulled out, the MNiTiH file was unscrewed counterclockwise and withdrawn from the canal, and the working length was recorded. Then, the X3 file of the PTN was inserted into the formed channel in a slow in-and-out pecking motion, with a 3-mm amplitude limit, to remove the gutta-percha up to the WL; also, gentle apical pressure was combined with a lateral brushing motion against the canal walls along the channel created previously. After three pecking movements, the instrument was removed from the canal and cleaned, and then the teeth were irrigated with 5 mL of 2.5% NaOCl. Similarly, the 35# 0.04–20# 0.03 MNiTiH file was used to gradually establish the glide path for PTN files using the crown-down manner until the 20# 0.03 MNiTiH file reached the apex. ProTaper Next files X3, X2, and X1 were sequentially used in a slow in-and-out pecking motion with 3 mm amplitude with a brushing motion against the root canal walls until the apical termination of the canal was reached. The X3 file was used for the removal of the coronal third of the root canal filling, followed by the X2 file for the middle third of the root canal, and the X1 file was used to reach the WL. Finally, the root canal of the apical third was reshaped to match the X3 file. When no obvious filling remnants in the root canal was observed under a dental microscope (Zumax; Suzhou; China), and there was no initial filling on the flutes of the X3 file, the retreatment procedure was completed.

### PTN combined with chloroform (PTN-CL)

Two drops of chloroform (0.5 mL) were placed on the root canal filling material for 2 min, and the PTN instrument was used in a slow in-and-out pecking motion with 3 mm amplitude with a brushing motion against the root canal walls. Specifically, ProTaper Next files X3, X2, and X1 were sequentially used in a crown-down manner. The X3 file was used to remove the coronal third of the root canal filling, followed by the X2 file for the middle third of the root canal. Finally, the X1 file was used to reach the WL. File penetration was carried out by using light apical pressure. Apical preparation was then performed with the X3 file. The solvent was refreshed when needed. Stop using chloroform when the X1 file was 2 mm from the apex. All instruments were driven by an X-smart Plus motor (Dentsply Maillefer; Ballaigues; Switzerland) at 300 rpm and 2 Nm torque as recommended in previous studies^19,20^.

The same irrigation protocol was used in all groups; between each instrument change, 2.5% NaOCl solution was delivered using a 5-mL syringe and a 30-G needle with a total of 20 mL 2.5% NaOCl delivered per canal. Then, 5 mL 17% EDTA was delivered to the area for 3 min, and a final rinse with 5 mL distilled water was performed. Each instrument was used for five specimens and then discarded. After the retreatment procedure, the canals were gently dried with paper points, and the specimens were subjected to micro-CT scanning with the aforementioned parameter settings.

### Root canal retreatment evaluation by micro-CT

All specimens were scanned at four-time points (before root canal preparation, after canal preparation, after canal filling, and after root canal retreatment), the obtained DICOM files of the scanned batches were coregistered in 3D using the Elastix rigid image registration module within 3D Slicer (v4.1.1) software (Harvard SPL; Boston; MA; USA). The acquired registered data were segmented with semiautomatic threshold-based segmentation to form a 3D canal and RFMs model for measurement by 3D Slicer. The RFMs region was set from the cemento-enamel junction to 0.5 mm from the apex of the root (entire canal). The volume of RFMs and the percentage of RFMs-covered areas in the entire canal were measured. The volume of RFMs of the total, apical third, and coronal two-thirds after retreatment was measured by 3D Slicer software. The measurement of the RFMs-covered surface was carried out as follows: both the surface of the region of interest (ROI) of the canal and the surface of intracanal residual filling materials were generated and exported to STL format in 3D slicer. The surface area of the canal wall in contact with RFMs was determined by calculating the area of the canal surface and the Boolean intersection (one voxel = 30 μm) in Geomagic Studio 2012 software (Raindrop Geomagic; Research Triangle Park; NC; USA). The surface was regarded as uncontacted when the distance between the canal surface and RFMs surface exceeded at least one voxel (24 μm); in that case, the Boolean intersection of the canal surface and the contacted surface area of RFMs was zero. All measurement work was performed by the same person. The percentage of the volume of RFMs and the area of RFMs were calculated using the equation below:

$$\frac{{{\text{Volume}}\;{\text{of}}\;{\text{RFMs}}}}{{{\text{Volume}}\;{\text{of}}\;{\text{canal}}}} \times 100\; = \;\% \;{\text{Volume}}\;{\text{of}}\;{\text{RFMs}}$$

$$\frac{{{\text{Canal}}\;{\text{surface}}\;{\text{area}}\;{\text{covered}}\;{\text{by}}\;{\text{RFMs}}}}{{{\text{Canal}}\;{\text{surface}}\;{\text{area}}}} \times 100\; = \;\% \;{\text{Area}}\;{\text{of}}\;{\text{RFMs}}$$

### Time and apically extruded debris

**Figure 2:**
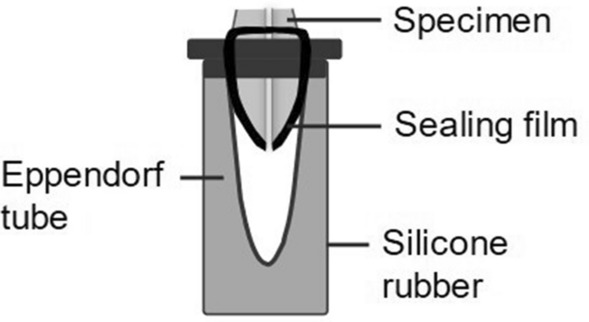
Schematic presentation of the apparatus for collecting apically extruded debris.

The time for each group of X1 files to reach the WL for the first time was recorded as T1, and the total time required for the retreatment to be completed was recorded as T2. The apically extruded debris was collected in an apparatus (Fig. 2) as previously described^18^. The surfaces of the apical 5-mm segment of specimens were wrapped with sealing film leaving the apical foramen and the coronal portion exposed. Each specimen was fixed on the lid of an Eppendorf tube through a precustom hole in which the apical 5-mm segment of the specimen was inside the tube and its coronal portion was outside. The space between the specimen and the Eppendorf tube was sealed with Top Dam (FGM; Joinville; SC; Brazil). When the retreatment procedure was completed, the specimens and the sealing film were removed. The Eppendorf tube was weighted using an electronic balance with 10^–4^ g accuracy (METTLER TOLEDO; Zurich; Switzerland) after the debris was dried, from which subtracting the weight of the empty Eppendorf tube, the weight of apically extruded debris was obtained.

### Statistical analysis

The Shapiro–Wilk and Levene test were used to evaluate the assumption of normality and the equality of variance among the datasets. Considering that the operating time, the amount of apical debris extrusion, and the percentage of volumes and covered canal surface area of the remaining filling materials were normally distributed, they were presented as the means and standard deviations. The independent-samples t test was then used to evaluate the percentage of volume and of covered canal surface area of RFMs, and analyze the difference in working time and weight of apically extruded debris between groups. SPSS software (Statistical Package for Social Science; SPSS, version 20.0, SPSS; Armonk; NY; USA) was used for statistical analysis, and the level of significance was set at 0.05.

## Results

### RFMs analysis

RFMs were observed in all experimental groups after root canal retreatment procedures. The mean volumes (%) of RFMs at different root canal portions and the mean percentage of the canal surface area covered by RFMs in different canal shape groups and in different instrument groups were shown in Table 1 and Table 2, respectively.

**Table 1:** Mean Percentage of Volume (mean ± standard deviation) and of Covered Canal Surface Area of RFMs in different canal shape groups.

	PTN-CL		PTN-MNiTiH	
	Round-canal	Oval-canal	Round-canal	Oval-canal
Coronal 2/3 volume (%)	2.4 ± 0.95*	6.3 ± 1.6*	1.7 ± 1.4*	3.14 ± 3.2*
Apical volume (%)	18.5 ± 3.8	19.25 ± 4.8	3.7 ± 2.1	7.6 ± 3.8
Total volume (%)	9.1 ± 4.7*	21.2 ± 7.5*	4.5 ± 1.3*	16.8 ± 5.5*
Coronal 2/3 area (%)	8.6 ± 1.9^#^	24.5 ± 7.9^#^	6.6 ± 4.7^#^	11.7 ± 8.9^#^
Apical area (%)	10.7 ± 3.1	26.8 ± 4.7	7.4 ± 3.1	17.6 ± 9.2
Total area (%)	14.5 ± 3.5^#^	22 ± 2.7^#^	9.2 ± 2.6^#^	15.9 ± 4.2^#^

*indicates a significant difference between different canal shape groups of the volume of RFMs (independent-samples t test, *P* < 0.05).

^#^indicates a significant difference between different canal shape groups of the covered canal surface area of RFMs (independent-samples t test, *P* < 0.05).

**Table 2:** Mean Percentage of Volume (mean ± standard deviation) and of Covered Canal Surface Area of RFMs in different instrument groups.

	Round-canal		Oval-canal	
	PTN-MNiTiH	PTN-CL	PTN-MNiTiH	PTN-CL
Coronal 2/3 volume (%)	1.7 ± 1.4^a^	2.4 ± 0.95^a^	3.14 ± 3.2^a^	6.3 ± 1.6^a^
Apical volume (%)	3.7 ± 2.1^b^*	18.5 ± 3.8^b^*	7.6 ± 3.8^b^*	19.25 ± 4.8^b^*
Total volume (%)	4.5 ± 1.3*	9.1 ± 4.7*	16.8 ± 5.5*	21.2 ± 7.5*
Coronal 2/3 area (%)	6.6 ± 4.7^#^	8.6 ± 1.9^#^	11.7 ± 8.9^#^	24.5 ± 7.9^#^
Apical area (%)	7.4 ± 3.2^#^	10.7 ± 3.1^#^	17.6 ± 9.2^#^	26.8 ± 4.7^#^
Total area (%)	9.2 ± 2.6^#^	14.5 ± 3.5^#^	15.9 ± 4.2^#^	22 ± 2.7^#^

Different superscript lowercase letters indicate a significant difference considering each group in the three-thirds of the teeth (independent-samples t test, *P* < 0.05).

*indicates a significant difference between different instrument groups of the volume of RFMs (independent-samples t test, *P* < 0.05).

^#^indicates a significant difference (*P* < 0.05) between different instrument groups of the covered canal surface area of RFMs (independent-samples t test, *P* < 0.05).

Between the groups of the same root canal shape, the mean percentage of the total volume of RFMs in samples in the PTN-MNiTiH groups was less than that in samples in the PTN-CL groups (*P* < 0.05); this significant difference was also observed in the apical third (*P* < 0.05). For all groups, the mean volume of RFMs in the apical third of the root canal was significantly greater than that in the coronal two-thirds of the root canal (*P* < 0.05).

When comparing the effect of different root canal shapes in each technique, no matter which method was used, the mean percentage of the total volume of RFMs in samples in the round-shaped groups was less than that in samples in the oval-shaped groups (*P* < 0.05); this significant difference was also observed at coronal 2/3 (*P* < 0.05). However, no significant difference was observed in the apical third of samples in the different root canal shape groups (*P* > 0.05).

**Figure 3:**
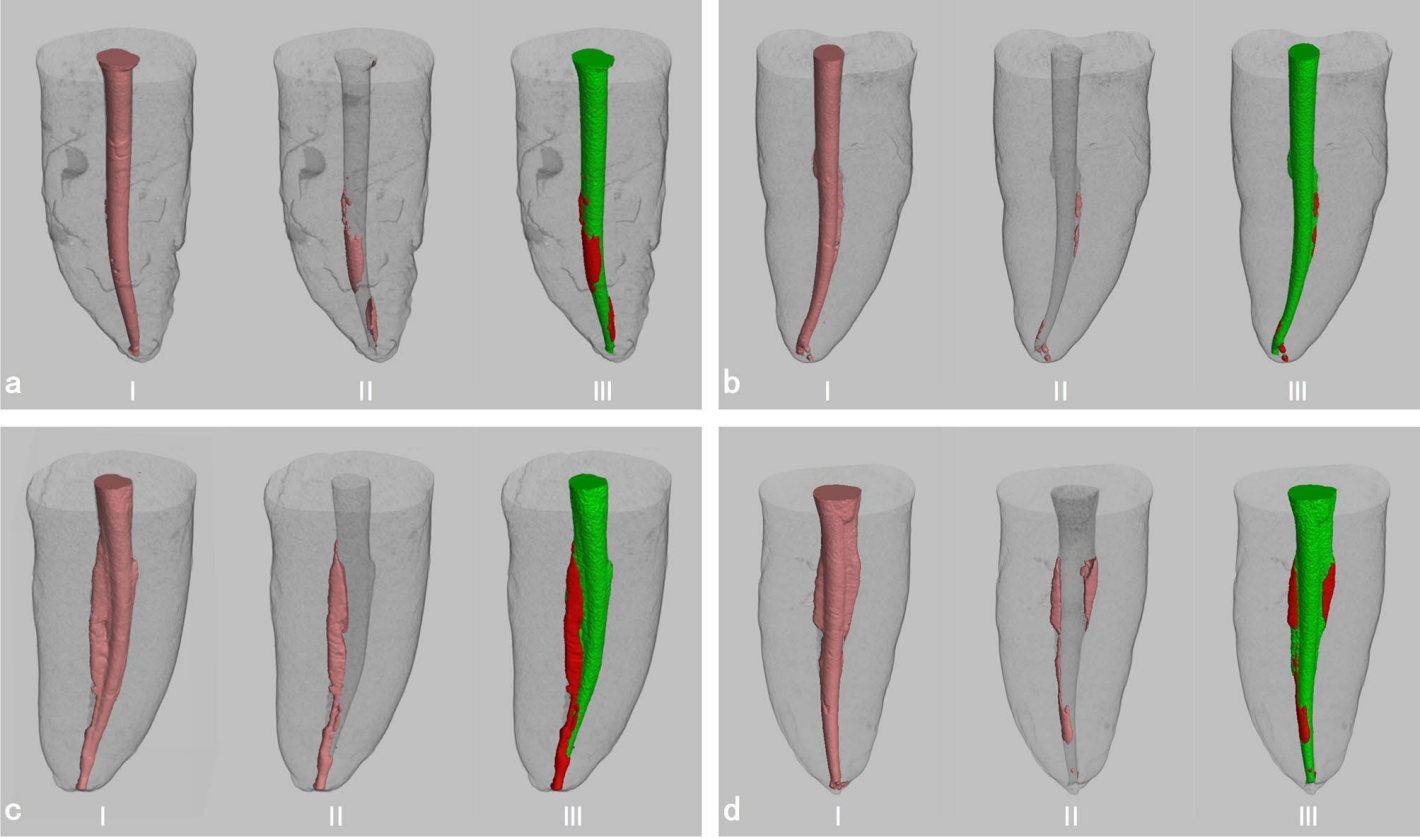
Representative samples of reconstructed 3D micro-CT images from each group (**a**) R-PTN-CL Group (**b**) R-PTN- MNiTiH Group (**c**) O-PTN-CL Group (**d**) O-PTN- MNiTiH Group) showing the filling materials (pink) after obturation (I), the remaining filling materials (pink) after retreatment with different methods (II), and the canal surface without covering by RFMs (green) and covered by RFMs (red).

The canal surface area covered by RFMs in samples in the groups with the different root canal shapes was significantly less both at the coronal two-thirds and the apical third in samples in the PTN-MNiTiH groups (*P* < 0.05). For samples in the oval-shaped canal groups, the surface area covered by RFMs in the coronal two-thirds was larger and the total untreated area was greater (*P* < 0.05) in samples in the PTN-CL group than in samples in the PTN-MNiTiH group (*P* < 0.05). For samples in all groups, there was no significant difference between the apical third and the coronal two-thirds for the surface area covered by RFMs. Figure 3 shows micro-CT images of representative specimens from each group before and after retreatment.

### The operating time of the two methods during retreatment

The mean operating times during root canal retreatment are represented in Table 3. There was no significant difference in either the time required to reach the working length (T1) (*P* > 0.05) or the time required to achieve satisfactory gutta-percha removal (T2) (*P* > 0.05) in all groups.

**Table 3:** Weight of Apically Extruded Debris in Milligrams (mean ± standard deviation) and Time Used for Instrumentation in seconds.

Group	R-PTN-CL	O-PTN-CL	R-PTN- MNiTiH	O-PTN- MNiTiH
T1 (reach the working length) (s)	263.00 ± 22.72	268.50 ± 23.11	256.67 ± 15.63	279.67 ± 27.95
T2 (Total operating time) (s)	396.50 ± 29.45	520.00 ± 36.33	433.33 ± 24.33	525.83 ± 22.02
extruded debris (mg)	3.7 ± 1.8^a^	4.2 ± 1.6^a^	1.58 ± 1.0^b^	1.6 ± 0.7^b^

Different superscript letters indicate statistical difference (*P* < 0.05).

### The extruded apical debris of the two methods

The mean values and standard deviations for the amount of extruded apical debris during root canal retreatment in samples in all groups are listed in Table 3. Between samples in the groups with the same root canal shape, the amount of extruded apical debris produced by the PTN-MNiTiH groups was less than that of the PTN-CL groups (P < 0.05). However, no statistically significant differences between samples in different root canal shape groups in which the same instruments were used were identified (*P* > 0.05).

## Discussion

Complete removal of the filling material remnants in the root canal is essential for thorough root canal irrigation and disinfection during root canal retreatment. Previous studies have shown that many filling materials remain in the root canal after filling materials are removed from the root canal of incisors by nickel-titanium instruments^21,22^. The additional use of organic solvents can soften gutta-percha, making it easier for the nickel-titanium instruments to be screwed into the filling materials, reducing the occurrence of instrument separation, dissolving the filling materials in irregular areas, and increasing the removal efficiency of the filling materials. However, their use increases the difficulty of root canal cleaning^23^ and has certain carcinogenicity^24^. To the best of our knowledge, there is no report on the use of nickel-titanium rotary instruments combined with MNiTiH files during root canal retreatment. Therefore, the present study was designed to evaluate the efficiency of the strategy of gutta-percha removal by nickel-titanium rotary instruments combined with MNiTiH files.

The results showed that less volume and a smaller covered area of RFMs were found in the MNiTiH file groups compared to the chloroform groups. Hence, the null hypothesis was rejected. MNiTiH files are root canal retreatment instruments made of M-wire nickel-titanium alloy, which is characterized by a large taper and superelasticity. With a large taper, it can be screwed into the filling materials to have a chance to pull out the filling materials in large pieces. Even if the filling materials cannot be directly pulled out, the channel established step-by-step by screwing MNiTiH files into the gutta-percha can effectively prevent instrument separation when the PTN is used for repreparation in a crown-down manner. Traditional hand stainless steel Hedström files are too rigid and prone to instrument separation, especially in curved canals.

As mentioned before, chloroform is often used to soften gutta-percha to make it easier for instruments to enter filling materials. However, the effectiveness of chloroform in cleaning root canals has been suggested to be limited in previous studies^12,25^, which is consistent with our research. The total area covered by RFMs and the volume of RFMs at the middle third of the root canal in samples in the PTN-CL groups were significantly higher than those in samples in the PTN-MNiTiH file groups. This result may be explained by the fact that softened gutta-percha has more possibility to be squeezed into the irregular area of the root canal and covers the root canal surface in the form of the membrane^26^ and thus increasing the difficulties of root canal cleaning.

This study also found that there was no significant difference with respect to the time to reach the working length and the time to complete root canal repreparation between samples in the PTN-MNiTiH file groups and the PTN-CL groups. However, the amount of extruded apical debris was reduced after the channel was established by MNiTiH files in samples in the different root canal morphology groups, which can effectively reduce the incidence of postoperative pain after root canal therapy^27,28^. This finding was consistent with Pawar et al*.*^29^, who found that spinning MNiTiH files into filling materials and pulling them out together reduced the possibility of extruding the debris out of the apical foreman.

Micro-CT is a noninvasive imaging technique with high resolution. This approach has been widely used in previous research on root canal retreatment, and the volume and the percentage of RFMs can be evaluated in three dimensions^6,30^. Biofilm can adhere to RFMs and increase the difficulty in canal disinfection. Additionally, RFMs can cover the canal surface, affecting the efficacy of canal irrigation and intracanal medications on the canal wall. Therefore, the untreated area covered by RFMs has a more significant impact on root canal retreatment, increasing the risk of reinfection. At present, the commonly used method for measuring the untreated area covered by RFMs is the measurement after splitting the roots longitudinally to evaluate the canal walls microscopically or with radiographs^31^. Nevertheless, this method is destructive, not three-dimensional, and there may be a risk of underestimating root canal wall cleanliness. Currently, few studies have discussed the area of the root canal covered by RFMs. Theoretically, for RFMs with the same volume, the larger the covered root canal area is, the more dentin tubules will be blocked. Thus, the effect of root canal irrigation and medication will be compromised. In our study, another important finding was that the area covered by RFMs in the circular canal was smaller than that in the oval canal, which was in line with our expectations. Because the cross-section of root canal repreparation instruments was round and did not match the shape of oval root canals, RFMs in buccolingual areas of root canals could not be contacted. Therefore, more RFMs were found in these areas. The surface area of samples covered by RFMs in the PTN-MNiTiH file groups was less than that of samples in the PTN-CL groups. The observed difference may be attributed to the lack of solvents in the PTN-MNiTiH file groups. The filling materials did not form a sticky film covering the root canal surface, so the contact area was smaller.

In line with previous studies^6,7^, the results of this experiment also showed that no matter which method was used and no matter what root canal morphology was, the filling materials in the root canal could not be removed entirely, and most RFMs were located in the apical third^30,32^. One possible reason for this result is that the anatomic variations are often most remarkable in the apical third; therefore, this finding underlines the debridement limitation of the currently available retreatment technology facing a complex root canal anatomy. These irregular areas cannot be contacted by the instrument, which highlights the limitation of the current instrument in that it cannot match complex root canal anatomical structures.

Samples in the round canal groups had less RFMs than samples in the oval canal groups, which is consistent with our expectations. This could be explained by the fact that PTN rotary files machine the root canal into a form with a round cross-section; therefore, substantial untouched areas may be left on the buccal and lingual sides of a flat root canal. It may be possible to further explore the removal efficiency of filling materials through some auxiliary methods, such as ultrasound instruments and apical enlargement. One of the purposes of this study was to investigate the effect of the morphology of the instrumented root canal on root canal retreatment in mandibular incisors, so straight root canals were selected. In this study, canals with curvatures higher than five degrees were excluded since the root canal curvature was positively correlated with nickel-titanium instrument fracture^33^. It was reported that in moderately and severely curved root canals, the instrument separation was attributed to a single overloading of the rotating instrument, and was independent of gradual degradation caused by fatigue.

Under the conditions of the present study, establishing a channel by screwing MNiTiH files into filling materials and pulling them out together may be a promising method for root canal retreatment, but further research remains to be carried out to confirm and validate these findings. Moreover, some additional measures can be used to further increase the removal effect on filling materials.

## Conclusion

Under the limitations of this in vitro study, none of the tested methods was able to completely remove the root canal filling materials. The two retreatment protocols showed similar performance in operation time. However, the PTN system combined with MNiTiH can remove root canal filling materials more efficiently and reduce the extruded apical debris compared with the use of the PTN system combined with chloroform. The volume of remnant root filling materials with a round-root canal system was significantly less than that observed in an oval-root canal system. More studies should be conducted to find the perfect combination of instrumentation to enhance material removal during endodontic retreatment of anatomically complex teeth.

## Data Availability

The data that support the findings of this study are available from the corresponding author upon reasonable request.
